# Positional Release Technique Versus Muscle Energy Technique for Patients With Nonspecific Low Back Pain With Facet Joint Restriction: A Pilot Randomized Comparative Study

**DOI:** 10.1155/prm/9964889

**Published:** 2026-03-24

**Authors:** Azzam Alarab, Rinad Daoud, Mohammed Moustafa Hegazy

**Affiliations:** ^1^ Department of Physiotherapy, Faculty of Post-Graduate Studies and Research, Palestine Ahliya University, Bethlehem, State of Palestine; ^2^ Department of Physical Therapy, College of Applied Medical Sciences, Prince Sattam Bin Abdulaziz University, Al-Kharj, Saudi Arabia, psau.edu.sa; ^3^ Department of Physical Therapy for Musculoskeletal Disorders and Its Surgery, Faculty of Physical Therapy, Cairo University, Cairo, Egypt, cu.edu.eg

## Abstract

**Objective:**

This study, being a pilot research, explored the comparative short‐term results, as well as the feasibility, of using positional release technique (PRT) and muscle energy technique (MET) in nonspecific low back pain sufferers who also exhibited a presumption of a restriction in the facet joint.

**Materials and Methods:**

For the purposes of the present pilot randomized comparative study, 36 patients between the ages of 25 and 50 years, all presented by an orthopedic surgeon with the diagnosis of NSLBP, were selected. These patients were randomly assigned equally to two groups using a computer‐generated randomization list. Group A patients were subjected to PRT, while Group B patients were subjected to MET. Before commencing each treatment, a 15‐min hot pack was administered universally. Pain intensity was also measured using the visual analogue scale (VAS), while functional measures were determined using the Roland‐Morris Disability Questionnaire. Three treatments per week over four weeks were administered.

**Results:**

Both interventions were found to be significant in reducing experimental pain from the beginning to the end of the study. After the PRT intervention, the VAS scores showed an average decrease of 1.5 points from 8.6 to 7.1. In the same manner, the VAS scores showed an average decrease of 5.66 points from 9.0 to 3.3 after the MET intervention was conducted. Concerning the RMQ scores in the same manner as the VAS of both PRT and MET intervention variables in the study, the PRT group experienced an average decrease of 1.05 in the RMQ scores from 9.3 to 8.2. After the MET intervention in the study was conducted in the same manner as the PRT intervention, the RMQ scores showed an average decrease of 9.05 points from 11 to 2.5.

**Conclusion:**

Based on this pilot RCT, the results indicate that the short‐term outcomes of patients with NSLBP who also have facet joint restrictions were more significantly improved through the use of the MET technique in comparison with PRT. Further research is necessary to confirm the potential of the MET technique to reduce pain associated with patients with NSLBP who have facet joint restrictions.

**Trial Registration:** ClinicalTrails.gov.identifier: NCT07165249

## 1. Introduction

Low back pain (LBP) refers to pain, soreness, or discomfort in the area between the lower ribs and the hips. It may arise from various causes such as muscle strain, injury, postural dysfunction, or underlying medical conditions [1]. LBP is a widespread global issue, with prevalence varying by region, population characteristics, and study methodology. Nevertheless, it affects a large proportion of the population at some point in life, with lifetime prevalence rates reported between 60% and 80% [2, 3].

Nonspecific low back pain (NSLBP)—pain not attributed to a specific pathology—is recognized as having considerable social and economic consequences. Individuals often experience muscle tension, reduced mobility, postural alterations, and emotional distress [4]. Early physiotherapeutic intervention may be beneficial, as symptoms can worsen over time and interfere with daily functioning and overall quality of life [5]. Physiotherapy offers evidence‐based strategies that may help manage NSLBP effectively [6]. These typically involve a combination of therapeutic exercise, manual therapy, education, and pain management approaches aimed at reducing symptoms, improving function, and preventing recurrence [7].

Several physiotherapy techniques are used to achieve these goals. Treatment strategies commonly combine targeted exercise programs, manual interventions, patient education on posture and movement, and individualized plans to address muscular imbalances and promote active recovery [2].

Among the manual therapy approaches, the positional release technique (PRT) and the muscle energy technique (MET) have attracted attention for treating LBP. PRT, including approaches such as strain–counterstrain, focuses on reducing pain and muscle tension by placing the affected tissues in a position of comfort to facilitate relaxation. Therapists identify tender points and adjust the patient’s posture to relieve strain and potentially restore muscle alignment [8].

MET is an active technique that requires patient participation. It is proposed to address musculoskeletal problems by having the patient perform controlled muscle contractions against resistance, which may help reduce muscle tightness and potentially improve joint movement. Physiotherapists, chiropractors, and other clinicians often use MET to assist in managing movement restrictions and muscle imbalances [9].

The efficacy of both PRT and MET could perhaps vary in view of the unique qualities that the individuals to be treated with these interventions possess [10]. The comparative study done involving both interventions was limited to individuals with chronic NSLBP and those with suspected facet joint pathology during NSLBP attacks. The target individuals for the pilot study with suspected facet joint pathology were based on the characteristics of the pain experienced in NSLBP.

This study’s selection of participants with suspected facet joint dysfunction was based more on clinical evaluation than imaging verification. To determine facet joint involvement, clinicians frequently rely on particular clinical signs, the patient’s medical history, and physical examination results, such as localized pain, pain that worsens with particular movements, and limited spinal mobility. In initial or research settings, imaging is not always required or feasible, even though it can offer conclusive confirmation. Given that these characteristics may imply a likely involvement of the facet joints even in the absence of imaging confirmation, patients displaying clinical characteristics consistent with facet joint dysfunction were thus included.

This randomized comparative pilot study was performed to examine the short‐term effects, feasibility, and safety of PRT as compared to MET based upon pain and functional outcomes of patients suffering from NSLBP who had clinical presentations of facet joint dysfunction. A preliminary answer to this may aid as a basis for an eventual randomized clinical trial.

## 2. Materials and Methods

### 2.1. Participants

This randomized comparative study was conducted at Al‐Maqassed Hospital in East Jerusalem between September 2023 and February 2024. Participants were adults aged 25 to 50 years with a clinical diagnosis of chronic NSLBP lasting more than three months. Patients were referred from the hospital’s orthopedic department to the outpatient physiotherapy unit for further evaluation and treatment.

Inclusion criteria consisted of individuals experiencing NSLBP, with or without radiating foot pain. Patients suspected of facet joint dysfunction based on clinical examination (e.g., localized lumbar pain aggravated by extension and rotation) were eligible, although no imaging‐based confirmation was used. Exclusion criteria included a history of spinal surgery, vertebral fracture, osteoporosis, inflammatory or metabolic bone disease, and diagnosed spondylolisthesis.

Considering the exploratory nature of the current research and the lack of previous direct comparative studies with such a research question among the investigated group of patients, the present investigation had a pilot design with a randomized comparative trial approach, including the assessment of the feasibility and safety aspects, and a preliminary estimation of the expected outcomes. A total group of 36 patients was selected based on the convenience sampling approach. The current results indicate that the designed pilot trial would have a high level of power with the detected differences in changes in the VAS. The mean difference equals 4.16 units with a pooled SD of 1.52. Within the approach with *α* = 0.05 and *n* = 18.0 in each group, the power equals 99.9%. However, the results indicate that the designed pilot trial would have been powered only to detect very large differences. A reanalysis with a plausible minimal clinically important difference (MCID) with an estimated change of 2.0 units on the VAS with an assumed SD = 1.5 resulted in a power of about 65.0%.

Randomization was performed using an off‐site, computer‐generated sequence, with allocation concealment ensured by sealed opaque envelopes. However, the treating physiotherapist was not blinded to group assignment, and a single therapist delivered both interventions, introducing potential performance bias. Additionally, outcome assessor blinding was not implemented.

### 2.2. Intervention Protocol

Participants were randomly assigned to one of two groups, each receiving 3 physiotherapy sessions per week over four weeks. Adherence to the intervention protocol was monitored through session attendance logs; participants who missed more than two sessions were excluded from final analysis.

Both groups received a 15‐min hot pack application to the lumbar region before manual therapy as a standardized cointervention for muscle relaxation.

Group A (PRT): Patients lie prone for the application of the hot pack. PRT was then applied to the erector spinae muscles. The therapist identified tender points and passively positioned the patient into a position of comfort, typically involving lateral trunk flexion toward the symptomatic side. Each session involved three 90‐s holds per tender point. PRT procedures followed standardized positioning principles as described in previous literature [8].

Group B (MET): Following the hot pack application, patients were assessed for segmental dysfunction via palpation of lumbar transverse processes in the prone position. MET was applied using isometric contractions aimed at correcting lumbar rotational or side‐bending dysfunctions. Each contraction was held for 7–10 s, followed by a passive stretch. The technique was repeated for 3–5 cycles per session. The MET protocol followed established clinical guidelines [9].

To ensure intervention fidelity, both PRT and MET had standardized protocols. All of the physiotherapists were trained in advance and employed structured checklists for each session to ensure consistency. There were weekly intersession calibration meetings to monitor protocol adherence and reduce variability between participants. These measures were taken to enhance the reproducibility and reliability of the study’s findings.

### 2.3. Outcome Measures

#### 2.3.1. Primary Outcome Measures Included

• Pain intensity, assessed using the visual analog scale (VAS)
• Functional disability, assessed using the Roland‐Morris Disability Questionnaire (RMQ)

Both were collected at baseline and after the four‐week intervention period.

The slump test and FABER test were used only during the initial screening phase to exclude specific causes of LBP (e.g., neural tension, hip joint pathology). As they were not used to assess treatment effectiveness, their detailed procedures are omitted here for brevity.

### 2.4. Data Analysis

Data were analyzed using SPSS Version 25.0. Descriptive statistics (mean ± SD for continuous variables, and frequencies with percentages for categorical variables) were used to summarize participant characteristics, including age, gender, and employment status. The distribution of continuous outcome variables was assessed using the Shapiro–Wilk test. For variables that followed a normal distribution, namely, the VAS scores and age, parametric tests were applied. Baseline comparisons of age between groups were examined using the independent samples *t*‐test, while within‐group pre–post changes in VAS were analyzed using the paired samples *t*‐test.

For variables that did not follow a normal distribution, including the RMQ scores and categorical demographic variables such as gender and employment status, nonparametric tests were applied.

Between‐group comparisons of RMQ scores were assessed using the Mann–Whitney *U* test, while within‐group pre–post changes were examined using the Wilcoxon signed rank test. Microsoft Excel was used for data entry and visualization.

**FIGURE 1:**
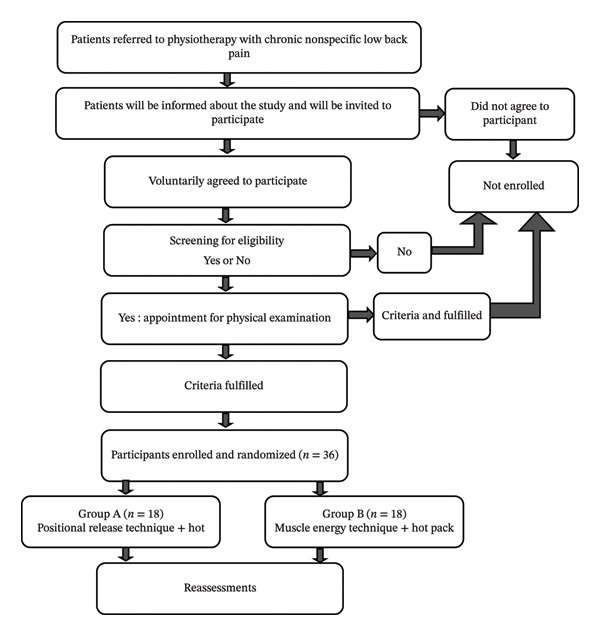
Recruitment process and flow through the study.

A CONSORT‐compliant flow diagram was developed to illustrate the recruitment and progression of participants through the study. The standardized CONSORT flowchart template from the EQUATOR Network was followed. Figure 1 illustrates the recruitment process and flow through the study.

## 3. Results

The study included 36 participants randomly divided into two equal groups (Group A = PRT; Group B = MET). The demographic distribution is presented in Table 1, which compares age, gender, and employment status across both groups. Independent samples *t*‐tests and chi‐square tests confirmed no statistically significant differences between groups in demographic characteristics (*p* > 0.05), indicating comparable baseline profiles (Table 1).

**TABLE 1:** Comparison of demographic characteristics between groups.

Variable	Group A (PRT) (*n* = 18)	Group B (MET) (*n* = 18)	*p* value
Age^∗^, years	36.3 ± 8.77	37.1 ± 9.58	0.80
Gender (*n*)			0.75
Male	10	11	
Female	8	7	
Employed (*n*)			0.69
Yes	14	15	
No	4	3	

*Note:* Chi‐square for gender and employment.

^∗^Independent sample *t*‐test for age.

Improvement in VAS scores in Group A and Group B was statistically significant. For Group A, PRT, there was a reduction in average VAS from 8.6 ± 0.8 to 7.1 ± 0.8. Also, there was a reduction in VAS of 1.5 points (95% CI: −2.11 to −0.90; *p* < 0.001; Table 2). However, this difference was not seen at a level that reached the MCID (approximately 2.0 points), which is generally accepted for the VAS in patients with chronic LBP. The scores on RMQ decreased in Group A from 9.3 ± 2.7 to 8.2 ± 2.6 (mean change: −1.05; 95% CI: −1.16 to −0.94; *p* < 0.001; Tables 2 and 3). Improvements were again statistically significant.

**TABLE 2:** Comparison of VAS scores for Group A sample before and after PRT.

Group A PRT		Mean	Standard deviation	*t* value	*p* value^∗^
VAS	Pre	8.6	0.85	5.3	< 0.001
	Post	7.1	0.83		

Abbreviation: VAS, visual analog scale.

^∗^Paired sample *t*‐test.

**TABLE 3:** Comparison of RMQ scores for Group A sample before and after PRT.

Group A PRT		Mean	Standard deviation	*Z* value	*p* value^∗^
RMQ	Pre	9.3	2.7	−4.14	< 0.001
	Post	8.2	2.6		

*Note:* RMQ, Roland‐Morris Low Back Pain and Disability Questionnaire.

^∗^Wilcoxon signed‐rank test.

In Group B (MET), the mean VAS improved substantially from 9.0 ± 0.76 to 3.3 ± 1.68 yielding a mean reduction of 5.66 points (95% CI: −6.42 to −4.21; *p* < 0.001), and RMQ decreased from 11.5 ± 3.16 to 2.5 ± 1.65 corresponding to a mean change of −9.05 (95% CI: −11.17 to −8.66; *p* < 0.001) (Table 4).

**TABLE 4:** Comparison of Group B sample before and after MET.

Group B (MET)		Mean	Standard deviation	*t* value	*p* value^∗^
VAS	Pre	9.0	0.76	15.30	< 0.001
	Post	3.3	1.68		

RMQ	Pre	11.5	3.16	12.04	< 0.001
	Post	2.5	1.65		

*Note:* RMQ, Roland‐Morris Low Back Pain and Disability Questionnaire.

Abbreviation: VAS, visual analog scale.

^∗^Paired sample *t*‐test.

Between‐group analyses demonstrated statistically significant differences favoring MET for both pain reduction and functional improvement (*p* < 0.001 for VAS and RMQ; Table 5).

**TABLE 5:** Mann–Whitney *U* test to examine the differences between the two groups.

	Group A PRT				Group B MET				*U-*score	*p* value^∗^
	Mean difference ±SD	95% CI	Mean rank	Total rank	Mean difference +SD	95% CI	Mean rank	Total rank		
VAS	−1.50 ± 1.20	−2.11 to −0.90	27.0	486	−5.66 ± 1.57	−6.42 to −4.21	10.0	180	9.00	< 0.001
RMQ	−1.05 ± 0.23	−1.16 to −0.94	27.5	495	−9.05 ± 3.19	−11.17 to −8.66	9.5	171	9.0	< 0.001

*Note:* RMQ: Roland‐Morris Low Back Pain and Disability Questionnaire.

Abbreviations: SD, standard deviation; VAS: visual analog scale.

^∗^Mann–Whitney *U* test.

## 4. Discussion

This pilot study offers initial comparative evidence on two different forms of physical therapy techniques for the handling of NSLBP with clinical characteristics of facet joint limitations: the MET and the PRT. Collectively, the data support the fact that both forms of therapy produced statistically significant in‐group outcomes pertaining to handling NSLBP with clinical characteristics of facet joint limitations. A prominent trend occurred in relation to both forms of therapy; nevertheless, a more definitive trend showed positive in‐group outcomes in comparison of PRT and MET therapies for treating with clinical characteristics of facet joint limitations. This is shown by the fact that the MET form of therapy had a reduction on the VAS score of −5.66 in comparison with the PRT form of therapy of −1.5; in comparison with the RMQ, there is evidence of a more definitive reduction in favor of MET.

Therefore, the extent of the difference observed between the two groups of patients can also be considered large, with a standardized value of 1.09 for the VAS outcome measures and 1.27 for the RMQ outcome measures. It should also be noted, in this pilot study, great caution must be exercised when interpreting findings based on these values and their corresponding. *p* values, as the reduced variability typical of pilot studies elevates the risk of inflated effect estimates and an increased likelihood of Type I error.

A further critical level of interpretation lies with contextualization against such measures of clinical significance. Where chronic LBP is concerned, a decrease of 2 points on a scale of 100 mm on a VAS is generally considered to represent the MCID that is expected to have meaning to a patient and thus constitutes a positive change in pain perception for such an individual. This highlights an additional level of differentiation with respect to understanding such patient therapy outcomes. In this way, we might suggest that the PRT group here has not necessarily benefited in terms of statistical significance with respect to pain perception levels.

This trend of results favoring MET finds a degree of concordance with the existing body of literature, though it is not always homogeneous. For example, Memon et al., in a comparative trial of dissimilar design, also reported MET to be somewhat more effective than PRT in the management of LBP [11]. Furthermore, the relevance of MET for targeting facet joint dysfunction is indirectly supported by works such as that of Wahyuddin et al., who reported positive outcomes when MET was combined with lumbar stabilization exercises for patients with pain of suspected facet joint origin [8]. A proposed mechanistic rationale for the apparent advantage of MET may relate to its fundamental operational principle. As an active technique, MET requires voluntary patient contraction followed by relaxation, a process theorized to facilitate neuromuscular re‐education, induce postisometric relaxation in hypertonic muscles, and potentially enhance proprioceptive awareness and segmental motor control [12, 13].

This active engagement might further hold particular significance for the management of the abnormal motor behaviors and functional losses commonly seen within a chronic condition of NSLBP. In the case of PRT, the approach is understood within the parameters of being a “passive modality,” whereby the therapist brings the patient into a position that produces an “ease of tissue,” with the concomitant reduction in “reflexogenic muscle guarding” that can contribute to the patient’s condition of discomfort [14]. While useful for the important function of influencing the patient’s condition of discomfort itself, this approach might lack the “engagement‐related motor‐learning component,” which could shed more significant functional benefit for the chronic condition under discussion. Mechanisms such as these remain “theoretical constructs,” in accordance with the findings of the present study and the literature that has addressed these factors thus far.

However, it is of critical importance to weigh these optimistic findings with a balanced assessment of the overall evidence base and the substantial methodological limitations of this investigation. In fact, a recent systematic review with a meta‐analysis conducted by Santos et al. [2], whereby authors analyzed aggregated findings of 19 RCTs, found that MET resulted in a small but significant effect on pain reduction for chronic NSLBP and a moderate effect on subacute pain but lacked significance on functional disabilities. This is an obvious contrast with the strong functional gains that have been demonstrated here and serves to illustrate the overall variability with which manual therapy research is often conducted and interpreted.

While the limitations associated with the current study are many and directly impact the overall interpretation of the study results, perhaps the greatest consideration is the actual size of the study population utilized within the current investigation (*n* = 36), which was not based upon any a priori statistical considerations. Thus, the study is able to detect only arduously large differences. Another consideration is the actual study design itself and the resultant bias which the authors report an absence of blinding for both the therapeutic intervention and the outcome assessment. As a result of the subjective nature of the reported results utilizing the VAS and RMQ analyses themselves, there is a resultant expectation bias which may have favored the more interactive PERT procedure over the passive PRT procedure that is utilized [15]. Other aspects that need to be mentioned in relation to their deficiencies include using only one therapist, which limits external validity; having a short intervention and follow‐up period, which limits understanding of long‐term result recurrence; and having used a clinical judgment of facet joint involvement instead of image evidence to create heterogeneity in subject inclusion criteria.

Therefore, it is evident that a clear framework for the trajectory of additional research is identified with regard to these limits and the preliminary nature of the findings. Certainly, to validate or refute these findings in totality, a definitive randomized controlled trial is indicated. This trial will need to be preceded by accurate calculations regarding sample size in accordance with a realistic difference of interest to be measurable in this setting. Methodological rigors will need to be added to this process to increase efficacy with regard to increased use of blinded outcome assessors (and participants if appropriate), utilization of multiple treating therapists to increase generalizability of findings in totality, and inclusion of extended outcome measures to be assessed at 3, 6, and 12 months in totality. Future studies might also usefully investigate whether PRT and MET act synergistically when combined in a treatment paradigm or how they compare to other cornerstone interventions for NSLBP, such as structured exercise therapy [5].

With regard to the conclusions of this pilot study being generalized to the larger population of patients suffering from NSLBP, this author would state the follwing: While this pilot study provided a promising initial indication of the potentially superior benefits to pain reduction and functional outcomes of MET relative to PRT in the scope of patients with NSLBP as described herein, such initial indications must nevertheless be viewed as having been generated as part of a larger backdrop of strongly limiting methodological constraints.

## 5. Conclusion

The pilot randomized comparative study presents preliminary findings for the short‐term use of PRT and MET for patients with NS‐LBP and signs of facet joint involvement. While statistically different improvements were noted for patients undergoing MET and PRT interventions on pain intensity and function disability measures, trends from the small sample size suggest that MET may be more effective in alleviating pain intensity and function disability. The improvement noted from PRT was statistically different but failed to reach the MCID for the pain intensity outcome.

However, these findings are preliminary and must be interpreted with considerable caution due to the methodological limitations of this study, which include small sample size, lack of blinding, short duration, and risk of bias. The results should be considered as providing a hypothesis that MET may be beneficial in the short term for this patient population—a hypothesis to be tested—and not as a superiority finding. Therefore, MET may be considered as a potentially useful component within a broad, patient‐centered management strategy for NSLBP, but not as a stand‐alone recommendation based on this evidence. The main value of the current study is to determine the feasibility of the protocol and provide preliminary data on the potential impact of the treatment in order to inform the design of a future definitive randomized controlled trial to allow the evaluation of the effectiveness of these manual therapy techniques.

**Nomenclature**
PRT: Positional release technique
MET: Muscle energy technique
NSLBP: Nonspecific low back pain
VAS: Visual analogue scale
RMQ: Roland‐Morris Questionnaires
LBP: Low back pain
FABER: Flexion Abduction External Rotation

## Author Contributions

Conceptualization, Rinad Daoud and Mohammed Moustafa Hegazy; data curation, Rinad Daoud and Mohammed Moustafa Hegazy; formal analysis, Azzam Alarab; investigation, Rinad Daoud and Azzam Alarab; methodology, Rinad Daoud and Mohammed Moustafa Hegazy; project administration, Mohammed Moustafa Hegazy; resources, Azzam Alarab; software, Azzam Alarab; supervision, Mohammed Moustafa Hegazy; validation, Rinad Daoud and Mohammed Moustafa Hegazy; visualization, Azzam Alarab; writing–original draft, Rinad Daoud; writing–review and editing, Azzam Alarab.

## Funding

No funding was received for this study.

## Ethics Statement

This study was approved by the Institutional Ethical Committee of Palestine Ahliya University (Approval No. CAMS/PTBR/3/124/2024). All procedures were conducted in accordance with the ethical standards of the 2008 Declaration of Helsinki. Prior to data collection, written informed consent was obtained from all participants.

## Conflicts of Interest

The authors declare no conflicts of interest.

## Data Availability

The data that support the findings of this study are available on request from the corresponding author. The data are not publicly available due to privacy or ethical restrictions.
